# Integrative Forensic Genetics, Biochemical, and Histological Methods for Reconstructing Biological Profiles from Aged Human Skeletal Remains

**DOI:** 10.3390/genes17030258

**Published:** 2026-02-25

**Authors:** Irena Zupanič Pajnič, Tamara Leskovar

**Affiliations:** 1 Institute of Forensic Medicine, Faculty of Medicine, University of Ljubljana, Korytkova 2, 1000 Ljubljana, Slovenia; 2 Centre for Interdisciplinary Research in Archaeology, Department of Archaeology, Faculty of Arts, University of Ljubljana, 1000 Ljubljana, Slovenia; tamara.leskovar@ff.uni-lj.si

**Keywords:** aged skeletal remains, human identification, biological sex, forensic DNA intelligence, FIGG SNP genotyping, multi-isotope analysis, methylation-based age prediction, tooth cementum annulation

## Abstract

The reconstruction of biological profiles from aged or degraded human skeletal remains represents a major challenge in both forensic and bioarcheological contexts, particularly when conventional identification approaches fail. Recent advances in molecular genetics, biochemical and histological analyses, and biomolecular anthropology have substantially expanded the range of information that can be recovered from compromised remains. This review synthesizes current integrative approaches combining genomic analyses, stable isotope investigations, epigenetic age estimation, proteomic sex determination, and complementary histological techniques to infer sex, ancestry, kinship, age, diet, mobility, and geographic origin. Genetic methods, including next-generation sequencing (NGS), enable increasingly robust inference even from highly degraded samples. Stable isotope analyses provide insights into dietary patterns and mobility, while DNA methylation markers improve age estimation accuracy. Tooth cementum annulation (TCA), although a histological rather than molecular method, contributes an additional chronological indicator within an integrative analytical framework. Rather than treating these approaches independently, this review proposes a multidisciplinary perspective in which complementary datasets collectively support biological profile reconstruction. Integrative interpretation enhances identification potential and provides more nuanced life-history reconstructions, demonstrating the value of combining molecular, biochemical, and histological evidence in forensic and archaeological investigations.

## 1. Introduction

Following death, individuals may become unrecognizable for various reasons, particularly when the postmortem interval is prolonged, making identification a prerequisite for burial and the resolution of legal and property matters. Rapid and reliable identification, therefore, has both humanitarian and legal significance. In cases involving unidentified skeletal remains with no available information about potential missing persons, conventional profiling of short tandem repeat (STR) markers is insufficient, as comparison with antemortem reference samples or putative relatives is not possible [1]. Under such circumstances, investigative leads can be generated by constructing a comprehensive biological profile using molecular genetic approaches, including the determination of sex, biogeographical ancestry, externally visible characteristics, uniparental lineages, extended kinship analyses, and searches within genetic genealogy databases to identify potential relatives. In parallel, multi-isotope analyses can provide complementary and independent information on an individual’s geographic origin, dietary patterns, physiological stress, as well as migration history and mobility. The combined interpretation of genetic and isotopic data allows investigators to significantly narrow the range of possible identities, develop focused investigative strategies, and substantially increase the probability of successful identification of missing persons. The present review does not restrict “biochemical methods” exclusively to isotopic analyses. Stable isotope analysis is discussed primarily in relation to diet and mobility reconstruction rather than chronological age estimation. However, biochemical age indicators such as racemization or protein cross-linking represent additional molecular pathways that may complement genetic and epigenetic approaches when suitable substrates are preserved. In this preservation-based framework, tooth cementum annulation (TCA) is situated alongside, rather than in competition with, molecular strategies, reinforcing the central argument that methodological complementarity, not exclusivity, provides the most robust basis for reconstructing biological profiles from aged skeletal remains.

Molecular genetic identification has become an exceptionally effective tool for the identification of missing persons and victims of mass disasters [2,3]; however, DNA preservation is strongly influenced by both the environmental context in which human remains are recovered and the duration of exposure to adverse conditions [4], meaning that DNA extracted from aged skeletal material is typically highly degraded and present in low quantity and quality, often further compromised by the presence of PCR inhibitors [5]. In addition, the high risk of contamination with modern human DNA represents a major limitation, as skeletal remains may be exposed to contemporary DNA from individuals involved in excavation, handling, or laboratory processing [6]. To minimize the risk of false-positive results arising from contamination, strict protocols for contamination prevention and authentication of compromised DNA must be implemented [7,8,9]. Specimen handling and DNA extraction should be performed in dedicated pre-PCR facilities in which no post-PCR work has ever been conducted [7], ideally equipped with HEPA-filtered hoods to prevent the introduction of exogenous DNA. All laboratory procedures should be carried out using appropriate protective clothing, and workspaces must be routinely decontaminated with bleach and UV irradiation [7]. Negative extraction controls should be processed in parallel with bone samples to monitor potential contamination from reagents and laboratory consumables [10]. Whenever possible, genetic profiles of excavators, anthropologists, and laboratory personnel included in the elimination database should be generated and verified to ensure that they do not match the profiles obtained from skeletal remains. Furthermore, multiple independent DNA extractions should be performed from each specimen, and concordant results should be obtained to confirm authenticity [7]. The DNA extraction protocol itself is critical for maximizing DNA yield and quality [11], and procedures based on complete demineralization combined with efficient DNA purification are essential to release DNA from hydroxyapatite and to remove PCR inhibitors commonly present in soil-derived bone samples [12]. Finally, before downstream genetic analyses, both the quantity and quality of recovered DNA should be assessed using quantitative PCR (qPCR) to ensure the suitability of the extracts for reliable genetic typing [13].

It is well established that skeletal elements differ in their suitability for genetic analyses because DNA preservation varies considerably among bones [14,15,16,17], a phenomenon that is particularly pronounced in aged skeletons that have been exposed to prolonged postmortem degradation. DNA preservation is also influenced by the chronological age of individuals [18]. A substantial body of research has demonstrated that the petrous portion of the temporal bone consistently provides the highest DNA yields, owing to its exceptional hardness and density, which make it more resistant to physical, chemical, and biological decay processes [4,19,20,21]. This bone displays distinctive structural and compositional properties, including a macrostructure dominated by thick cortical bone with minimal trabecular content, a highly mineralized apatite matrix, and an extensive system of vascular canals [22,23]. The superior preservation of DNA in the petrous bone has been attributed to its high bone density, limited remodeling activity, and elevated concentration of osteocytes, which collectively contribute to the long-term protection of endogenous DNA [24,25]. Consequently, specialized and detailed protocols for sampling and extracting material from the cochlear region of the temporal bone have been developed to maximize DNA recovery for genetic analyses [26]. Dental cementum provides a viable alternative to petrous bones for DNA extraction, enabling high-yield recovery via nondestructive methods that preserve tooth integrity for subsequent morphological analysis [1,27]. Environmental factors also influence DNA preservation in petrous bones, as demonstrated in comparative studies of different archeological sites [28]. In addition to environmental factors, post-excavation storage conditions of skeletal samples must be considered, as comparative analysis revealed significant differences in DNA preservation between freshly excavated bones and those stored in museum depots [29].

Skeletal remains from both forensic and ancient samples usually yield low DNA quantities and quality, are susceptible to PCR inhibitors, and carry a significant contamination risk [30,31]. In ancient DNA (aDNA) research, traditional PCR-based methods have recently been replaced by advanced techniques capable of sequencing entire genomes [32]; however, these are much more expensive than forensic methods and require specialized laboratories with highly skilled personnel. On the other hand, many PCR-based massively parallel sequencing (MPS) kits using targeted amplicon strategies have become commercially available in forensics [33,34], allowing for the generation of sequencing data at relatively low costs. When optimized, forensic molecular genetics methods are applicable to ancient skeletal material for tasks such as sex determination, lineage assessment, phenotyping, ancestry estimation, and kinship analysis—integrating with anthropological and archeological data to explore key questions about human history. Genetic profiling has identified notable historical figures [35,36], and autosomal and Y-chromosomal STR analyses have clarified kinship in medieval remains [37]. Our research group has also applied forensic-developed techniques to ancient subadult skeletons for genetic sex determination [38], first-degree kinship analysis via STR profiling of four individuals from a 5th–6th century burial site [39], and predictions of eye and hair color [40,41].

Isotope analyses of human skeletal remains have become an established and powerful approach for reconstructing aspects of individual life history, including diet, mobility, provenance, and physiological stress, in both forensic and archeological contexts [42]. In contrast to DNA, isotopic information is embedded in both the organic (collagen) and mineral (bioapatite) components of bones and teeth and reflects long-term interactions between the individual and their environment. However, the reliability of isotope-based reconstructions is strongly dependent on tissue preservation and post-mortem alteration. Diagenetic processes affecting bone collagen and bioapatite can modify original isotopic signals, particularly in remains exposed to prolonged burial, fluctuating soil chemistry, or extreme environmental conditions [43,44]. As a result, rigorous pretreatment protocols and quality control criteria are essential to assess collagen integrity, mineral preservation, and potential contamination before analysis [45,46,47].

Despite these limitations, isotope analyses are often more resilient to environmental degradation than DNA and can yield informative results even when genetic material is highly fragmented or absent. Different skeletal tissues record isotopic information over distinct temporal scales, with tooth enamel preserving childhood signals and bone reflecting later-life averages due to continuous remodeling, allowing life-course reconstruction that is not accessible through genetics alone [48,49]. Multi-isotope approaches combining light stable isotopes (H, C, N, O, S, Zn) with radiogenic lead and strontium isotope ratios further enhance interpretive power by integrating dietary, ecological, and geographic information [50,51]. In archeological research, isotope analyses have been widely applied to address questions of subsistence strategies, migration, social differentiation, and demographic structure, while in forensic investigations, they provide independent investigative leads when conventional identification methods are limited or unavailable. When integrated with molecular genetic data and osteological evidence, isotope analyses therefore represent a critical complementary approach for constructing multidimensional biological profiles from aged skeletal remains.

Unlike prior reviews that examine forensic genomics, isotope geochemistry, epigenetic age estimation, dental histology, or proteomics as isolated methodological domains, this article proposes a preservation-stratified integrative framework for reconstructing biological profiles from aged skeletal remains. Rather than treating analytical approaches as hierarchical or competing, we conceptualize them as complementary modalities whose applicability is determined by biomolecular survival, temporal resolution, and inferential scope.

The novelty of this review lies in its operational synthesis: genetic, proteomic, biochemical, epigenetic, and histological methods are systematically organized according to preservation state and biological timescale within a unified analytical matrix. By explicitly linking molecular stability to forensic information yield and refinement level, we move beyond technique-centered summaries toward a structured interpretative model tailored to degraded skeletal material.

This preservation-sensitive perspective is particularly relevant in forensic casework, where molecular degradation, environmental exposure, and incomplete datasets are common constraints. Apparent discrepancies between methods are reframed as reflections of distinct biological targets and temporal windows rather than analytical inconsistency. Integrating these complementary signals within a structured preservation-based framework enhances robustness, transparency, and reproducibility in biological profile reconstruction.

## 2. Integrative Analytical Framework for Biological Profile Reconstruction

The growing diversity of analytical techniques available for the study of degraded skeletal remains necessitates a structured, integrative framework guiding method selection and interpretation. Rather than applying molecular, biochemical, and histological approaches independently, biological profile reconstruction benefits from a sequential and context-dependent analytical strategy. The first step involves evaluating skeletal preservation and biomolecular survival potential. Dense skeletal elements such as the petrous portion of the temporal bone and dental cementum frequently yield higher endogenous DNA content, whereas enamel and collagen preservation may instead favor proteomic or isotopic analyses. Preservation assessment, therefore, determines the feasibility and priority of subsequent analytical methods. When DNA preservation allows, genomic analyses provide foundational identification parameters, including biological sex, genetic ancestry, and kinship relationships. In cases of limited DNA survival, enamel proteomics, particularly amelogenin peptide analysis, may provide reliable sex determination independent of nucleic acids. Stable isotope analyses for life-history reconstruction contribute information regarding diet, mobility, and environmental exposure, reflecting long-term behavioral and geographic patterns. Epigenetic markers, especially DNA methylation profiles, enhance chronological age estimation, while TCA offers an independent histological age indicator that can complement molecular estimates.

For integration and interpretation of multimodal evidence, apparent discrepancies between analytical methods should not necessarily be interpreted as contradictions but as reflections of differing temporal, biological, and molecular resolutions. Genetic ancestry reflects deep population history, whereas isotopic signatures capture shorter-term signals of mobility and geographical residence. Similarly, divergences between methylation-based age estimation and histological age assessment may arise from differences in biological targets, tissue preservation, or intrinsic biological variability rather than analytical error. Integrative interpretation, therefore, requires evaluating each dataset within its temporal and biological context.

Interpretation of multimodal evidence, therefore, requires contextual evaluation of each dataset according to its preservation state, temporal sensitivity, and biological specificity. Because biomolecules degrade at different rates, DNA being generally more labile than proteins and mineral-bound collagen, method selection must be preservation-driven rather than hierarchical. Each analytical approach contributes distinct components of the biological profile (e.g., ancestry, age, diet, sex), and their integration strengthens inferential reliability while reducing interpretative bias. This preservation-sensitive, integrative framework promotes robust and transparent reconstruction of biological profiles in forensic casework, particularly when working with highly degraded or environmentally exposed skeletal material (see Table 1). Table 1 summarizes analytical options according to biomolecular preservation state and expected forensic information yield. “Temporal resolution represented” indicates the biological time window reflected by each method (e.g., inherited genomic signal, biological age at death, cumulative skeletal remodeling, or recent dietary history). “Level of refinement” refers to the degree of individual specificity achievable under optimal preservation conditions and should not be interpreted as absolute evidentiary certainty. Constraints listed reflect methodological, preservational, or interpretative limitations commonly encountered in forensic casework. The framework is intended to support preservation-informed analytical selection and integrative interpretation across complementary datasets.

## 3. Sex Determination

In the analysis of skeletal remains, sex estimation is a fundamental step in reconstructing an individual’s biological profile [52]. The reliability of macroscopic methods is highly dependent on the preservation and completeness of the remains, and such approaches enable only sex assessment rather than definitive sex determination, which requires the application of molecular genetic methods [53,54]. Our previous work demonstrated that genetic sexing can be successfully applied even to subadult skeletons, for which morphological assessment is considered unreliable, and that its success is independent of the age of the deceased child [38]. Forensic genetic analyses use various sex-informative markers located on the X and Y chromosomes, which are also applicable to ancient human remains. Some assays target both chromosomes, most notably the amelogenin test. In contrast, others are based solely on Y-chromosomal markers, such as qPCR assays amplifying Y-specific targets and Y chromosome single-nucleotide polymorphism (Y-SNP) typing. Among these, qPCR-based Y-target amplification is the most rapid and cost-effective method and is included in several commercially available forensic qPCR kits that quantify both autosomal DNA and male-specific DNA. The amelogenin assay, one of the earliest molecular approaches to sex determination, co-amplifies homologous regions on the X and Y chromosomes, targeting a short fragment in intron 1, and produces amplicons of approximately 100 bp that differ in length between the two [55]. It is incorporated into virtually all commercial human identification STR kits, enabling simultaneous genetic sex determination and individual identification through autosomal STR profiling [55]. A third sex-informative method is Y-SNP analysis, recently implemented in the ForenSeq Kintelligence kit—Qiagen (Hilden, Germany) [34]. Because forensic and archeological skeletal remains are typically characterized by low DNA quantity and high fragmentation, assays targeting short amplicons are particularly suitable, and all three of these tests amplify fragments shorter than 150 bp, making them appropriate for degraded samples. In low-template DNA contexts, male sex can be reliably confirmed only when all three tests produce positive results for Y-specific markers, whereas the absence of such signals does not allow unequivocal assignment of female sex because allelic dropout due to stochastic effects cannot be excluded [56].

In cases of poor DNA preservation, biological sex can still be determined through the analysis of amelogenin peptides preserved in dental enamel. Amelogenin-based sex determination from dental enamel represents a robust molecular approach for assessing biological sex in both forensic and archeological contexts, particularly when DNA preservation is poor. Amelogenin is the principal structural protein of tooth enamel and is encoded by homologous genes on the X and Y chromosomes (*AMELX* and *AMELY*), which differ in sequence length and peptide composition. During enamel formation, amelogenin peptides become entrapped within the mineral matrix and can persist long after DNA has degraded [57]. Sex determination is achieved by detecting sex-specific amelogenin peptides using mass spectrometry–based proteomic methods, most commonly liquid chromatography–tandem mass spectrometry (LC–MS/MS) [21,58]. Because enamel is the hardest and most diagenetically resistant tissue in the human body, amelogenin peptide analysis provides a highly reliable alternative for sex estimation when skeletal morphology is ambiguous or when genomic approaches are compromised. As such, enamel proteomics constitutes an important complementary tool within integrated biological profiling frameworks, bridging molecular genetics, osteology, and biochemical analyses in both forensic identification and bioarcheological research.

## 4. Forensic DNA Intelligence (FDI)

FDI leverages biological samples to produce investigative leads in criminal cases or human remains investigations where standard forensic STR profiling fails to individualize evidence from crime scenes or identify unknown, missing, or deceased individuals. This approach gained feasibility through the swift development of forensically pertinent markers beyond STRs, such as SNPs that reveal individual identity, biogeographical ancestry, and visible phenotypic traits. Forensic Investigative Genetic Genealogy (FIGG) represents the latest expansion of FDI tools [59]. Its proven success in resolving unidentified remains, missing persons cases, and cold violent crimes has spurred widespread adoption. FIGG typically involves generating SNP profiles via whole-genome arrays or sequencing from samples, followed by matching DNA segments against commercial genealogy databases. Historically, this required DNA quantities and qualities often unattainable from forensic specimens. The recently introduced ForenSeq Kintelligence kit (Qiagen) addresses these limitations with 10,230 SNPs—9867 (96%) dedicated to kinship inference—employing targeted amplicon sequencing optimized for low-input, inhibited, and degraded forensic samples [59]. High-density SNP genotyping enhances the detection of potential relatives in unidentified remains, cold cases, and innocent initiatives, as closer relatives share more inherited markers from recent common ancestors [60]. FIGG workflows entail uploading dense SNP profiles to public or law-enforcement-accessible genetic genealogy databases to pinpoint relatives, then constructing family trees to focus on lineages with missing or unaccounted members [61]. The GEDmatch PRO platform (available at: https://pro.gedmatch.com/, accessed on 27 December 2025) supports forensic SNP uploads and features the One-to-Many Kinship Tool, which matches unknown profiles against its vast database of voluntarily submitted DNA—primarily from direct-to-consumer providers like 23andMe, AncestryDNA, FamilyTreeDNA, Living DNA, and MyHeritage—based on shared DNA segments [62]. As the sole commercial solution, the Kintelligence Kit delivers robust FIGG alongside intelligence for sex determination, phenotyping, ancestry, lineage, and identity to aid in remains identification or evidence individualization. Ancestry-informative SNPs, which cluster within population groups and diverge across them, enable biogeographical origin estimation [63]. Principal component analysis (PCA) serves as a standard method, grouping individuals into ancestry clusters via two- or three-dimensional projections [64], readily distinguishing superpopulations such as European, African, and Asian. Phenotype prediction employs multinomial logistic regression (MLR) on trait-informative SNPs to forecast hair colors (brown, blond, red, black) and eye colors (brown, blue, intermediate) [65].

The ForenSeq Kintelligence kit (Qiagen), a single-panel assay, targets 10,230 SNPs—including 24 for phenotypic traits, 56 for ancestry, 94 for identity, 85 Y-chromosomal, 106 X-chromosomal, and 9867 for kinship inference—sequenced via the MiSeq FGx (Verogen, San Diego, CA, USA) system. It generates robust SNP profiles from inputs as low as 0.1 ng DNA [34] or even lower, as validated internally for forensic genetic genealogy applications [66], making it suitable for low-template samples like those from skeletonized bone remains [34]. Shorter amplicons (<150 bp) in the panel further boost success rates with degraded DNA, while the kit’s buffer formulation effectively counters common bone-derived inhibitors such as calcium, humic acid, microbial agents, and tannic acid. Evaluation by the Australian Federal Police’s National DNA Programme for Unidentified and Missing Persons, using human remains samples, confirmed high predictive precision for biological sex (via X/Y SNPs), genetic ancestry (via ancestry SNPs), and pigmentation traits like eye and hair color (via phenotypic SNPs). Kinship accuracy was verified by uploading SNP profiles from a 10-member family group to GEDmatch PRO, successfully identifying and categorizing relationships from first to fifth degree [34]. A separate validation with 12 family members (spanning first- to sixth-degree relatedness) affirmed the kit’s reliability for close ties, though sixth-degree links went undetected [59]. The ForenSeq Kintelligence MPS FIGG assay excels at classifying kinship up to fifth-degree relatives (e.g., great-great-grandparent to grandchild or first cousin once removed), achieving peak performance for first- through fourth-degree detections [67]. Unlike forensic STR profiling, which supports direct or close familial matching (typically first- and sometimes second-degree), this approach enables inference of remote relationships and generation of leads. Consequently, FIGG emerges as a valuable next step once standard STR database searches and conventional identification techniques prove insufficient.

## 5. DNA Methylation Analysis for Chronological Age Estimation

Epigenetic changes, particularly DNA methylation at cytosine–phosphate–guanine (CpG) sites, represent a promising molecular biomarker for estimating chronological age in human remains [68,69,70,71]. Unlike static genetic variation, DNA methylation patterns are dynamic and correlate strongly with age, reflecting cumulative environmental and physiological influences on the genome. Strong age correlations have been reported across blood, saliva, buccal cells, bones, and teeth, with mean absolute deviations (MAD/MAE) typically in the 2–6-year range when tissue-specific models are used [68,72,73,74]. In forensic contexts, methylation-based age estimation complements genetic identification by narrowing down potential identities when morphological methods are limited by preservation or taphonomic alteration, especially in skeletonized remains where soft tissues are absent [70,72,75].

Bones and teeth have received particular attention because they are frequently recovered years after death and retain DNA when other tissues have decomposed. In a bone- and tooth-specific study on Portuguese individuals, Correia Dias developed several age prediction models using bisulfite Sanger sequencing and a multiplex SNaPshot assay [72]. In bones, a six-CpG Sanger-based model targeting *ELOVL2*, *EDARADD*, and *MIR29B2C* explained 92.5% of age variation, with a MAD of 2.56 years, whereas SNaPshot-based dual-locus models using *FHL2/KLF14* yielded a MAD of 7.18 years [75]. For teeth, SNaPshot models combining *ELOVL2* and *KLF14* explained 76.4% of age variation with MAD 7.07 years, while single-CpG *FHL2* predictors were less accurate (MAD 11.35 years) [72]. A separate tooth study using bisulfite pyrosequencing of *ELOVL2*, *ASPA*, and *PDE4C* in 65 teeth reported significant age associations for multiple CpGs in *ELOVL2/PDE4C* and MAE around 4–6 years, with limited additional value from telomere length [73]. Other dental work confirms that *ELOVL2* and *EDARADD* methylation in whole teeth or tooth roots can predict age with MAEs of ~6–8 years across adulthood [73,76], and that cementum-rich root surface scrapings preserve informative *FHL2* methylation even in non-fresh, stored molars [77].

Genome-wide studies further support bone-specific epigenetic clocks. Gopalan et al. [78] analyzed 165 bone donors (living and deceased) using Illumina methylation arrays, identifying 108 age-associated CpGs and building a lasso-regression model that outperformed traditional morphometric methods in adult skeletons. Notably, two of the 37 CpGs retained in their final model mapped to *ELOVL2*, underscoring the cross-tissue robustness of this gene as an age predictor in bone as well as in blood, saliva, buccal cells, and teeth [68,73,78].

At the level of specific skeletal elements, Lee et al. developed an “epigenetic age signature” for postmortem ribs, analyzing eight CpGs in *ELOVL2*, *FHL2*, *KLF14*, and *FAM123C* by pyrosequencing. Their rib-specific model achieved R = 0.908 and MADs of 5.1 years in 81 rib samples, while a matched blood model for the same individuals performed slightly better (R = 0.927, MAD ~4.4 years). Cross-tissue application (blood model to ribs and vice versa) resulted in systematic bias, highlighting the importance of tissue-specific calibration [69]. Exploratory comparison of sternum, rib, and frontal bone samples from 12 decedents showed no significant differences in methylation levels or age estimates across these bone types, suggesting that at least some CpG panels may generalize across flat and cranial bones, though larger validation sets are needed [69]. Comparing skeletal tissues, cortical bone from ribs and long bone diaphysis tends to provide more precise age estimates (MAD ~3–5 years) than more porous trabecular bone, likely due to better DNA preservation and reduced surface-to-volume exposure to taphonomic processes [69,78]. Teeth, especially molars, generally yield slightly higher errors (MAE/MAD ~4–8 years) but remain valuable when cranial or long bones are unavailable [72,73]. Root-surface and cementum-focused approaches offer the additional advantage of preserving tooth morphology, which is important in medico-legal and archeological contexts [77].

The Visible Attributes through Genomics (VISAGE) enhanced tool represents a major step toward operational bone age estimation. Using a bisulfite multiplex PCR followed by MPS of 44 CpGs from eight markers, the VISAGE bone model employs six CpGs from *ELOVL2*, *FHL2*, and other genes and reaches a MAE of 3.4 years in bones, comparable to 3.2 and 3.7 years in blood and buccal cells, respectively [68]. The assay is optimized for low DNA amounts, making it suitable for forensic bone samples. Subsequent work has extended VISAGE-type panels and related SNaPshot tools to skeletonized material with very low DNA input. Recent advances demonstrate bisulfite-based success with ultra-low DNA inputs from skeletal remains, as shown in optimized VISAGE and SNaPshot protocols [68,69].

Postmortem and technical factors remain critical in skeletal applications. DNA from bones and teeth typically undergoes fragmentation, hydrolysis, and depurination over time, and bisulfite treatment further degrades templates. Nevertheless, multiple studies report that age-informative CpGs such as those in *ELOVL2*, *PDE4C*, *EDARADD*, and *MIR29B2C* are sufficiently stable in dense cortical bone, ribs, and dentin to support accurate age prediction even after extended postmortem intervals, as long as DNA preservation is moderate and fragment sizes allow successful amplification of short bisulfite-converted targets [69,73,78,79]. Although dentin has been successfully used for mitochondrial DNA recovery due to its association with odontoblast processes located at the pulp–dentin interface, nuclear DNA preservation within dentin is generally more variable and strongly dependent on post-mortem conditions. Consequently, dentin is often considered a secondary source for nuclear DNA compared with dental cementum or the petrous portion of the temporal bone [27]. VISAGE validation experiments have shown that carefully designed multiplex MPS panels can tolerate small DNA quantities and yield reproducible methylation values, but method-to-method bias and inter-laboratory variability still necessitate strict assay standardization and model training on data generated with the same platform [68,79].

Systematic reviews confirm that DNA methylation is currently the most accurate molecular method for age estimation in forensic practice, with typical MAEs of 3–5 years, compared to larger errors for telomere length or T-cell receptor excision circles [70]. *ELOVL2* emerges as the most consistently informative marker across tissues, including bone and teeth, and appears relatively robust to disease-related methylation changes [73,78,80].

Looking ahead, methylation-based age estimation in skeletal remains is likely to shift towards integrated, bone-optimized models trained on large, postmortem datasets, combined with low-input bisulfite MPS workflows such as VISAGE, and supported by AI/ML approaches that can exploit small CpG panels while controlling for degradation, tissue specificity, and population structure [68,78]. When combined with genetic, isotopic, and morphological evidence, such bone- and tooth-based clocks will substantially refine the biological profiles of unidentified decedents in forensic and humanitarian investigations.

## 6. Tooth Cementum Annulation (TCA)

TCA is a histological method for estimating chronological age based on incremental growth layers deposited in dental cementum. Unlike the genetic and biochemical approaches discussed elsewhere in this review, TCA represents a microscopic structural analysis rather than a molecular technique. It is included here because integrative biological profiling benefits from combining molecular and non-molecular age indicators that provide independent chronological information. TCA enables estimation of adult age at death based on the incremental deposition of acellular extrinsic fiber cementum on tooth roots, in which alternating light and dark bands are interpreted as annual growth increments [81,82,83]. Age is estimated by counting these increments and adding the mean eruption age of the respective teeth. The reliability of TCA has been robustly demonstrated in a large known-age validation study by Wittwer-Backofen et al. [84], which showed a strong linear relationship between TCA-derived and true chronological ages, with 95% confidence intervals generally within ±2.5 years when standardized preparation, imaging, and counting protocols were applied. Importantly, this study found no significant effects of sex, periodontal disease, or tooth type on age estimation accuracy. Subsequent research has confirmed that TCA can outperform conventional skeletal age indicators in adults, particularly where macroscopic methods yield broad or unreliable age ranges [82,85]. A comprehensive recent review by Perrone et al. [83] synthesizes these findings and highlights both the strengths and methodological challenges of TCA, including variation in sectioning techniques, microscopy, and criteria for increment interpretation, emphasizing the need for methodological standardization. When applied with careful specimen selection, replication, and integration within a multi-method age-estimation framework, TCA provides a valuable and independent line of evidence that complements other biological age indicators.

Although TCA is frequently cited as a promising method for estimating chronological age in adult individuals [85], it should not be interpreted as the dominant or universally applied dental histological technique in forensic anthropology. Other dentally based histomorphological approaches, most notably root dentin transparency methods such as the Lamendin technique, remain more routinely implemented in forensic casework due to their relative simplicity, lower laboratory requirements, and broader validation across population samples [86,87]. Root transparency-based approaches quantify age-related structural changes within dentin rather than incremental cementum deposition, and they are often favored in operational forensic settings where rapid, minimally destructive assessment is required. However, root transparency methods such as the Lamendin technique may be limited in cases of severe root surface erosion, advanced periodontal disease, apical fragmentation, or transparency saturation in elderly individuals [88]. Under such conditions, incremental cementum analysis (TCA) may offer an alternative avenue for age estimation when cementum microstructure remains preserved, and thin-section preparation is feasible.

Within the context of this review, TCA is therefore not presented as a methodological standard, but rather as one example of high-resolution histological aging that may be particularly informative under conditions of good dental preservation. Its inclusion reflects the broader aim of illustrating how distinct analytical domains, genetic, biochemical, proteomic, epigenetic, and histological, can contribute complementary information depending on tissue survival and investigative needs.

## 7. Isotope Analyses

Light stable isotope ratios of H, C, N, O, S, and Zn (δ^2^H, δ^13^C, δ^15^N, δ^18^O, δ^34^S, and δ^66^Zn) and radiogenic lead (^206^Pb/^204^Pb, ^207^Pb/^204^Pb, ^208^Pb/^204^Pb) and strontium isotope ratios (^87^Sr/^86^Sr) preserved in human tissues reflect the isotopic composition of an individual’s diet, drinking water, and local environment over different temporal scales [44,89,90]. In forensic contexts, isotope analyses provide independent information on dietary practices, physiological stress, residential mobility, and geographic provenance, thereby generating investigative leads when conventional identification approaches are limited [42,91,92]. When applied to archeological human remains, isotope analyses offer critical insights into past lifeways and social organization, including subsistence strategies, migration and mobility patterns, provenance, and population-level demographic variation [44,50]. Multi-isotope profiling of human remains typically focuses on organic components such as bone or dentine collagen, as well as mineral fractions including bioapatite in bones and teeth, each recording distinct aspects of an individual’s life history and enabling integrated life-course reconstruction [45,50,51,92].

Isotope ratios of different elements provide complementary information about diet, environment, and mobility. Carbon (δ^13^C) and nitrogen (δ^15^N) isotope ratios in human tissues are primarily used to reconstruct dietary practices, reflecting both the sources of dietary carbon and an individual’s position within the food web. Carbon isotopic values are incorporated into body tissues either directly through the consumption of plant foods or indirectly via animal products such as meat or dairy. Two key types of dietary information can be derived from δ^13^C values: the relative contribution of marine versus terrestrial resources and the consumption of C_3_- versus C_4_-based foods within terrestrial ecosystems [93,94]. Nitrogen isotope ratios (δ^15^N) exhibit systematic enrichment with increasing trophic level, with higher δ^15^N values generally observed in consumers with greater intake of animal protein [93].

Zinc isotope ratios (δ^66^Zn) have recently emerged as a valuable dietary proxy in bioarcheological and forensic research, particularly for reconstructing trophic level and the relative contribution of animal versus plant protein. Zinc isotopes are measured in the mineral fraction of skeletal tissues and are therefore not affected by collagen degradation. Experimental, modern, and archaeological studies have demonstrated systematic zinc isotope fractionation along the food chain, with higher trophic levels characterized by lower δ^66^Zn values due to physiological fractionation during digestion and metabolism [51,95]. As a result, δ^66^Zn values in human skeletal tissues have been shown to correlate with the proportion of animal protein in the diet and to distinguish dietary patterns that may be difficult to resolve using collagen-based isotope systems alone, particularly in C_3_-dominated terrestrial ecosystems.

In contrast, sulfur isotope ratios (δ^34^S) primarily reflect local geological and environmental conditions and are particularly useful for distinguishing between marine, coastal, and terrestrial resource use, as well as between different geological settings [96]. Sulfur isotopic signatures enter terrestrial and aquatic food webs through soils, bedrock weathering, and atmospheric inputs, with marine sulfate characterized by relatively uniform and elevated δ^34^S values compared to the more variable signatures of terrestrial environments [97]. Because sulfur undergoes minimal trophic-level fractionation, δ^34^S values in human tissues largely preserve the environmental sulfur signal of consumed foods, making this isotope system especially effective for identifying coastal influence and distinguishing between otherwise isotopically similar terrestrial diets [96].

Hydrogen (δ^2^H) and oxygen (δ^18^O) isotope ratios provide complementary information related to the isotopic composition of meteoric water consumed through drinking water and food. Because δ^2^H and δ^18^O values in precipitation vary systematically with geographic factors such as latitude, altitude, and distance from the sea, their incorporation into body tissues can contribute to the reconstruction of individual provenance and residential mobility, particularly when integrated with other isotope systems [98,99].

Lead isotope ratios emerged as a valuable complementary tool in forensic investigations, extending the capability of multi-isotope provenance beyond traditional stable isotope systems. Lead has four stable isotopes (^204^Pb, ^206^Pb, ^207^Pb, ^208^Pb), and the ratios of radiogenic ^206^Pb, ^207^Pb, and ^208^Pb relative to ^204^Pb vary according to local geology, anthropogenic input, and historical sources, creating geographically distinctive isotopic signatures. Although lead isotope applications in forensic anthropology have been less common than those of strontium or oxygen, they have been used effectively to differentiate regional Pb signatures in human tissues and to contribute additional geographic discrimination when other isotopic systems overlap [89,100,101].

The strontium isotope ratio (^87^Sr/^86^Sr) in the local environment varies spatially as a function of underlying geology, reflecting the age and composition of bedrock and its weathering products. Because bioavailable strontium is incorporated into human tissues through a combination of drinking water and dietary intake and undergoes negligible biological fractionation, ^87^Sr/^86^Sr values in bones and teeth reflect the geological signatures of the landscapes in which individuals resided. As a result, strontium isotope analysis can be used to distinguish between local and non-local individuals within a given region and to identify patterns of residential mobility and migration [50,102,103].

Isotope values in an organism’s tissues are also influenced by changes in physiology associated with nutritional and physiological stress, such as food scarcity, pregnancy, illness, or metabolic disruption. Because organisms strive to maintain homeostasis, isotopic signatures reflect the balance between dietary inputs and the metabolic demands of energy production, growth, and tissue repair. Shifts between anabolic and catabolic metabolic states can therefore alter isotope values independently of dietary composition. During periods of nutritional stress, such as fasting, malnutrition, or disease, insufficient protein and caloric intake lead to a predominance of catabolic processes, in which endogenous body tissues are metabolized. This results in the recycling of nitrogen within the body and a corresponding increase in δ^15^N values in tissues. Conversely, when nutritional requirements are met, and anabolic processes dominate, nitrogen is preferentially routed toward tissue synthesis, leading to lower δ^15^N values [104,105].

Stable isotope analyses can be applied to a range of biological tissues; however, in skeletonized remains, bones and teeth are typically the only tissues preserved and available for analysis. Because skeletal tissues form and remodel at different times and rates during the life course, they provide the opportunity to investigate isotopic signatures associated with distinct life stages. Most skeletal elements begin forming in utero but undergo continuous remodeling throughout life, a process involving the replacement of older tissue with newly formed bone [106]. This remodeling results in the progressive incorporation of isotopic signals reflecting more recent dietary and environmental inputs, while earlier signals are gradually overwritten. Consequently, isotope values derived from bone represent a time-averaged signal spanning several years before death, with the temporal resolution depending on the specific skeletal element as well as the individual’s age, sex, and health status [49,107]. In contrast, teeth also begin forming in utero but do not remodel once their formation is complete. As a result, tooth enamel and primary dentine collagen preserve isotopic information specific to the period of tooth development, typically corresponding to childhood and adolescence [108]. This fundamental difference between bone and dental tissues allows isotope analyses to distinguish between early-life and later-life dietary and environmental signals and to reconstruct changes across the life course.

Isotope analyses have a long-standing tradition in ecology and geology and have been widely adopted in archeology and physical anthropology to investigate past environments and human lifeways. In recent years, these approaches have also become increasingly integrated into forensic science, particularly in the investigation of unidentified human remains. By combining multiple isotope systems, multi-isotope analyses enable more refined reconstructions of dietary practices, physiological stress, residential mobility, and migration across different stages of the life course. Recent forensic applications have demonstrated that multi-isotope analysis constitutes a powerful complementary investigative tool, capable of generating probabilistic assessments of geographic provenance, mobility patterns, and migration histories when conventional identification methods yield limited or inconclusive results [101,109,110,111].

## 8. Discussion

The integration of molecular genetic analyses and stable isotope techniques represents a transformative advance in forensic identification science. The ForenSeq Kintelligence kit (Qiagen), which employs MPS on the MiSeq FGx (Verogen) platform, enables the recovery of comprehensive SNP profiles from even highly degraded skeletal remains [34]. This technology not only enhances the ability to determine sex, ancestry, phenotype, and uniparental lineage, but also provides the foundation for forensic investigative genetic genealogy (FIGG) that can reveal extended kinship links up to the fifth degree [59,67]. Such high-resolution kinship inference is invaluable in cases where traditional STR profiling fails, particularly in the identification of missing persons and historical or conflict-related remains [61].

The synergistic integration of genetic data with isotope analyses enables a multidimensional reconstruction of an individual’s biographical history [51,89,96,103]. These isotopic markers record environmental and biological signatures that reflect both life-history processes and movement across landscapes. By integrating multiple isotope systems, such as δ^2^H and δ^18^O linked to drinking water and climate, δ^13^C and δ^15^N reflecting dietary composition and trophic level, δ^34^S indicating underlying geology and the use of marine, coastal, or terrestrial resources, δ^66^Zn providing additional resolution of animal versus plant protein intake, and ^206^Pb/Pb^204^, ^207^Pb/Pb^204^, ^208^Pb/Pb^204^ and ^87^Sr/^86^Sr indicating geological provenance, it becomes possible to distinguish between early-life and later-life residence and to infer mobility and migration with greater precision. When combined with genetic information, multi-isotope datasets substantially enhance the interpretive framework available for reconstructing individual life histories, particularly in cases where conventional identification approaches yield limited results.

The integration of molecular genetics and isotopic datasets enables the construction of a composite biological profile that bridges genetic identity with environmental and life-history context. While SNP data can inform on biological sex, ancestry, and familial relationships, isotope analyses provide complementary evidence of geographic mobility, dietary practices, and physiological changes experienced over the course of an individual’s life. Together, these independent yet synergistic lines of evidence allow for a more nuanced reconstruction of personal biography than either approach alone. This integrative framework has proven particularly valuable in humanitarian forensic contexts, where reconstructing both identity and life history of deceased individuals supports legal investigations and contributes to the identification process, thereby facilitating resolution and emotional closure for affected families [42,112].

The inclusion of multiple skeletal elements, such as teeth, ribs, and femora, ensures isotopic coverage across distinct phases of the life course, encompassing childhood, adulthood, and the final years before death. This multi-tissue approach enables the assessment of temporal changes in diet and residential history, as different skeletal elements integrate isotopic signals over different timescales [49,107]. When combined with genomic markers, isotopic data allow for the reconstruction of individual biographical trajectories with an unprecedented level of temporal and contextual resolution. Such integrative evidence not only narrows the pool of potential identity matches but also enhances the robustness and inferential confidence of identity reconstruction in cases involving unidentified human remains.

Furthermore, the ethical and privacy implications of FIGG use must be acknowledged. The voluntary nature of public genetic databases such as GEDmatch PRO requires rigorous compliance with data protection standards and informed consent [62]. Future forensic practice should adopt transparent frameworks that balance investigative utility with individual privacy rights. Harmonizing these ethical standards internationally is crucial for the broader adoption of genetic genealogy and for maintaining public trust in forensic applications.

Multiple targeted methylation models have harnessed age-associated shifts at CpG sites in genes including *ELOVL2*, *FHL2*, *KLF14*, *PDE4C*, and *EDARADD* [74,113]. Horvath’s cross-tissue epigenetic clock, relying on 353 genome-wide CpGs, achieves mean absolute deviations of 3–4 years in living donors [71]. Forensic-tailored variants, such as the Forensic Epigenetic Age Estimation (FEAGE) framework, employ compact panels of top-performing CpGs amenable to bisulfite sequencing or pyrosequencing [114]. These approaches extend effectively to skeletal tissues, where methylation signatures persist across prolonged postmortem periods under moderate-to-good DNA preservation [115,116]. Cortical bone and dentin maintain reliable age signals at key loci, supporting chronological predictions from skeletal remains decades old. Integrating such epigenetic estimators with SNP-derived sex, ancestry, and kinship insights will sharpen biological profiles for unidentified cases, enable multidimensional reconstructions of identity, and surpass morphological limits in degraded contexts. As sequencing technologies advance, standardized methylation panels for degraded DNA will likely become routine in forensic laboratories, allowing more accurate, objective, and reproducible age estimation from skeletal remains.

Within an integrated biological profiling framework, TCA contributes a critical age-related dimension that complements genetic, isotopic, and osteological data in both forensic and archeological investigations [81,83,84,85]. Whereas molecular genetic analyses primarily inform on biological relationships, ancestry, and sex [32], sex determination may also be achieved through amelogenin peptide analysis of tooth enamel, providing a proteomic alternative in cases where DNA preservation is limited or absent [58,117], and isotope analyses elucidate aspects of diet, mobility, and environmental exposure [44,118]. TCA provides an independent estimate of chronological age in adulthood, thereby anchoring these datasets within a life-course perspective [82]. In forensic casework, this age information helps to constrain missing persons searches and refine comparisons with antemortem records, particularly when other age indicators yield broad or conflicting estimates [83,85]. In archeological contexts, TCA enhances demographic reconstruction by improving adult age-at-death estimates, which in turn strengthens interpretations of population structure, mortality patterns, and social organization [81,84]. When integrated with isotopic evidence derived from tissues formed at different times and with genomic markers of kinship and ancestry, TCA facilitates more precise reconstruction of individual biographies and population-level life histories. As such, TCA functions not as a standalone technique, but as a key component within a multidisciplinary framework for establishing robust and contextually informed biological profiles.

## 9. Conclusions

The construction of a detailed biological profile from aged skeletal remains is increasingly achievable through the convergence of molecular genetics, stable isotope analysis, DNA methylation profiling, TCA, and anthropological assessment. Advanced genomic tools, such as the ForenSeq Kintelligence kit, enable the recovery of informative SNP profiles from minute and highly degraded samples, supporting inference of sex, ancestry, phenotype, and extended kinship, while isotope analyses provide insights into biogeographical origin, mobility, dietary practices, and physiological stress over the life course. Within this integrative framework, DNA methylation–based age estimation and TCA offer complementary approaches to refining adult age-at-death, supplying independent chronological constraints that enhance the interpretive power of genetic and isotopic data. Together, these methods enable a multidimensional reconstruction of individual life histories, strengthening both forensic identification efforts and bioarcheological interpretations when conventional morphological indicators are limited or ambiguous.

Future directions should emphasize the continued refinement of SNP panels to enhance resolution for ancestry and phenotype prediction, the establishment of global reference isotope databases for provenance assessment, and the implementation of standardized integrative analytical pipelines. Collaborative frameworks between geneticists, isotope geochemists, and forensic anthropologists will be essential to realize the full potential of this multidisciplinary approach.

Ultimately, the combined use of molecular genetics, amelogenin peptides, stable isotope analyses, and chronological indicators such as DNA methylation profiling and TCA transforms skeletal remains into highly informative biological archives. The integration of different profiling methods has revolutionized the concept of biological profile in forensic science. By embedding genetic identity, environmental exposure, and age-related information within a single framework, such comprehensive profiling aids in solving contemporary forensic cases and contributes significantly to our understanding of human history, mobility, and adaptation. This multidisciplinary framework provides investigative leads that transcend traditional identification limits and enhance the accuracy and depth of personal reconstruction from skeletal remains.

The future of biological profile reconstruction does not reside in the continued expansion of isolated analytical techniques, but in their coordinated application within preservation-guided integrative frameworks. Aged skeletal remains are inherently heterogeneous, and biomolecular survival is uneven across tissues and environments. Consequently, methodological selection must be conditioned by preservation state rather than disciplinary preference. The structured integration of genetic, biochemical, proteomic, epigenetic, and histological evidence—each operating on distinct temporal and biological scales—allows complementary signals to be evaluated within their appropriate interpretive context. Such an approach enhances robustness, mitigates overinterpretation of single-method results, and improves transparency in forensic decision-making. By aligning analytical strategy with preservation realities and temporal resolution, integrative frameworks provide a more resilient model for reconstructing biological profiles from degraded human remains.

## Figures and Tables

**Table 1:** Preservation-guided integrative framework for reconstructing biological profiles in aged skeletal remains.

Biomolecular Preservation State	Recommended Analytical Strategy	Forensic Information Yield	Temporal Resolution Represented	Level of Refinement	Key Constraints/Caveats
**High endogenous DNA preservation**	Genome-wide SNP analysis/DNA methylation profiling	Sex, ancestry, kinship, phenotypic markers, epigenetic age	Lifetime (genetic)/biological age at death (epigenetic)	High individual specificity	Cost, contamination risk, population reference bias
**Moderate DNA preservation**	Targeted SNP panels/DNA methylation profiling	Sex, ancestry, kinship, phenotypic markers, epigenetic age	Lifetime (genetic)/biological age at death (epigenetic)	Moderate to high	Calibration population dependency, partial genomic coverage
**Low DNA preservation, proteins preserved**	Enamel proteomics (amelogenin-based)	Biological sex	Developmental (fixed during tooth formation)	High (binary trait)	Limited trait scope, destructive sampling
**Collagen and enamel were preserved**	Stable isotope analysis (C, N, Sr, O)	Diet, mobility, geographic origin	Years to decades before death	Moderate (probabilistic)	Environmental baseline requirement, indirect inference
**Teeth available (cementum intact)**	Cementochronology	Chronological age	Lifetime cumulative (annual increments)	Moderate to high (if preservation is good)	Histological variability, observer dependence
**Severely degraded biomolecules**	Histological/morphological assessment	Age-at-death estimation, remodeling indicators	Cumulative skeletal remodeling	Moderate to low	Broad error margins, population variation

## Data Availability

No new data were created or analyzed in this study.
